# Association of gamma‐glutamyl transferase concentrations with all‐cause and cause‐specific mortality in Chinese adults with type 2 diabetes

**DOI:** 10.1111/1753-0407.13399

**Published:** 2023-05-09

**Authors:** Haoyu Guan, Ke Liu, Xikang Fan, Hao Yu, Yu Qin, Jie Yang, Zheng Zhu, Chong Shen, Enchun Pan, Yan Lu, Jinyi Zhou, Jian Su, Ming Wu

**Affiliations:** ^1^ Department of Epidemiology, School of Public Health Nanjing Medical University Nanjing China; ^2^ Department of Epidemiology and Health Statistics, School of Public Health Southeast University Nanjing China; ^3^ Department of Non‐communicable Chronic Disease Control Provincial Center for Disease Control and Prevention Nanjing China; ^4^ Department of Chronic Disease Prevention and Control Huai'an City Center for Disease Control and Prevention Huai'an China; ^5^ Department of Chronic Disease Prevention and Control Suzhou City Center for Disease Control and Prevention Suzhou China

**Keywords:** cancer, cardiovascular disease, gamma‐glutamyl transferase, mortality, type 2 diabetes mellitus, γ‐谷氨酰转移酶, 2型糖尿病, 死亡率, 心血管疾病, 癌症

## Abstract

**Background:**

Evidence links gamma‐glutamyl transferase (GGT) to mortality in the general population. However, the relationship of GGT with all‐cause and cause‐specific mortality risk has been little explored in type 2 diabetes mellitus (T2DM) patients.

**Methods:**

We recruited 20 340 community‐dwelling T2DM patients between 2013 and 2014 in Jiangsu, China. Cox regression models were used to assess associations of GGT with all‐cause and specific‐cause mortality. Restricted cubic splines were used to analyze dose–response relationships between GGT and mortality. Stratified analysis was conducted to examine potential interaction effects by age, sex, smoking status, body mass index (BMI), diabetes duration, and dyslipidemia.

**Results:**

During a median follow‐up period of 7.04 years (interquartile range: 6.98–7.08), 2728 deaths occurred, including 902 (33.09%) due to cardiovascular disease (CVD), and 754 (27.58%) due to cancer. GGT concentrations were positively associated with all‐cause, CVD, and cancer mortality. Multivariable hazard ratios (HRs) for the highest (Q5) vs. the lowest quintile (Q1) were 1.63 (95% confidence intervals [CI]: 1.44–1.84) for all‐cause mortality, 1.87 (95% CI: 1.49–2.35) for CVD mortality, and 1.43 (95% CI: 1.13–1.81) for cancer mortality. Effect modification by BMI and dyslipidemia was observed for all‐cause mortality (both *p* for interaction <.05), and HRs were stronger in the BMI <25 kg/m^2^ group and those without dyslipidemia.

**Conclusions:**

Our findings suggest that, in Chinese T2DM patients, elevated serum GGT concentrations were associated with mortality for all‐cause, CVD, and cancer, and further research is needed to elucidate the role of obesity, nonalcoholic fatty liver disease, and lipids in this association.

## Graphical abstract

**Highlights**

- In people with type 2 diabetes, the risk of all‐cause, cardiovascular disease, and cancer mortality increased with higher gamma‐glutamyl transferase (GGT) concentrations.
- Stronger effects of GGT levels and all‐cause mortality were observed in the group with body mass index <25 kg/m^2^ and those with dyslipidemia.
- GGT could potentially be used to predict the risk of cardiovascular disease and cancer among people with type 2 diabetes.

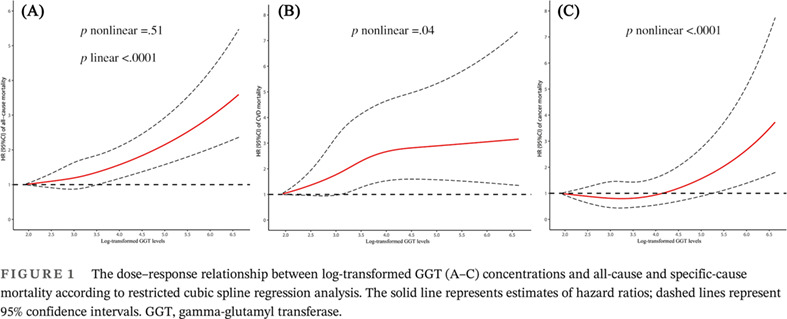

## 摘要

背景:有证据表明在一般人群中γ ‐谷氨酰转移酶(GGT)与死亡率相关。然而, 在2型糖尿病(T2DM)患者中, GGT与全因死亡率及病因特异性死亡风险的关系研究较少。

方法:本研究于2013至2014年在江苏招募了20,340例社区T2DM患者。采用Cox回归模型评估GGT与全因死亡率及病因特异性死亡率的关系。限制性立方样条用于分析GGT和死亡率之间的剂量反应关系。按年龄、性别、吸烟状况、体质指数(BMI)、糖尿病病程和血脂异常进行分层分析。

结果:在中位7.04年(四分位距:6.98 ~ 7.08)随访期间, 共2728例死亡, 其中902例(33.09%)死于心血管疾病(CVD), 754例(27.58%)死于癌症。GGT浓度与全因死亡率、CVD死亡率和癌症死亡率呈正相关。与最低五分位数(Q1)相比, 最高五分位数(Q5)的多变量风险比为全因死亡1.63 (95% CI: 1.44 ~ 1.84), 心血管疾病死亡1.87 (95% CI: 1.49 ~ 2.35)和癌症死亡1.43 (95% CI: 1.13 ~ 1.81)。观察到BMI和血脂异常对全因死亡率的影响(相互作用P均< 0.05), 并且在BMI < 25 kg/m^2^组和没有血脂异常的人群中多变量风险比更高。

结论:在中国2型糖尿病患者中, 血清GGT水平升高与全因死亡、心血管疾病死亡和恶性肿瘤死亡相关, 肥胖、NAFLD和血脂在其中的作用有待进一步研究。

## 1 INTRODUCTION

Diabetes mellitus is a chronic metabolic disorder that is rapidly becoming more common. According to the International Diabetes Federation, the global diabetes prevalence in 2021 was approximately 10.5% (536.6 million people), with the vast majority having type 2 diabetes mellitus (T2DM).^1^ The global prevalence of nonalcoholic fatty liver disease (NAFLD) in T2DM patients reached almost 60%, and the prevalence and incidence of cardiovascular diseases (CVD) have been found to be increased in individuals with NAFLD.^2^ Furthermore, NAFLD is a condition known to impact gamma‐glutamyl transferase (GGT) activity and mortality.^3^ Several prospective studies among general population have reported that elevated serum GGT concentrations are associated with a higher risk of CVD mortality.^4^, ^5^, ^6^ GGT is suggested to be a marker of metabolic and cardiovascular risk factors. Moreover, a meta‐analysis including 14 cohort studies suggested positive associations of serum GGT concentration with overall cancer risk.^7^ However, the relationship between GGT concentration and mortality risk has been little explored in T2DM patients.

Serum GGT is commonly used as a nonspecific marker of liver dysfunction and a biological marker of alcohol consumption.^8^ In addition to their utility in clinical practice, serum GGT concentrations have attracted interest primarily for their associations with CVD, diabetes, metabolic syndrome, and cancer. Prior studies showed that GGT concentration was elevated in people with T2DM, even in prediabetes.^9^ There is also strong evidence for a relationship between elevated serum GGT concentration and increased oxidative stress, which have been related to diabetes, cancer, and atherosclerosis and resulting cardiovascular events.^10^, ^11^, ^12^ A prospective study of 28 838 Finnish men and women showed that the associations between serum GGT concentration and coronary heart disease (CHD) risk were obviously stronger in T2DM patients than in the general population.^13^ However, contradictory results were observed in another analysis.^14^ In addition, studies have found regional differences between GGT concentrations and mortality. One meta‐analysis including 10 prospective studies reported that heterogeneity was reduced and nearly eliminated in most analysis after excluding two studies conducted in Asian populations.^15^ Therefore, future studies including more Asian populations will help in gaining insight into the nature of these processes. Based on a Chinese T2DM prospective cohort, this study explored the associations of serum GGT concentrations with all‐cause and cause‐specific mortality in T2DM populations.

## 2 METHODS

### 2.1 Study population.

Participants were enrolled from the Comprehensive Research on the Prevention and Control of the Diabetes (CRPCD) project in Jiangsu, China. In brief, the CRPCD project started in 2013, and 20 340 inhabitants were involved at baseline. A standardized questionnaire was used to collect information on demographic characteristics, self‐reported chronic disease history, and lifestyle factors. In addition, physical measurements and blood samples were taken at baseline. In the current analysis, we excluded 287 participants with important information lacking, 40 without GGT data, and 52 with chronic liver disease at recruitment. Therefore, a total of 19 961 participants were included in the final analysis.

### 2.2 Assessment of serum gamma‐glutamyl transferase concentrations.

Blood samples were collected in the morning after overnight fasting. All samples were analyzed in the laboratory at KingMed Diagnostics (Jiangsu Cultural Industrial Park, Nanjing, China). Serum GGT was measured by the enzymatic rate method (Roche Cobas C701, Roche Diagnostics (Shanghai) Ltd).

### 2.3 Ascertainment of outcomes

Participants were observed from the date of enrolment until 31 December 2020. Causes of death was obtained from the death certification system of the Jiangsu Provincial Center for Disease Control and Prevention. This system provides complete and accurate specific‐cause death data that have been medically validated. The primary outcomes of the current study included all‐cause mortality (A00‐Z99), CVD mortality (I00‐I79), and cancer mortality (C00‐C97). In addition, deaths due to CVD were divided into the following subgroups: CHD (I20‐I25) and stroke (I60‐I64). Cancer deaths were divided into the following subgroups: esophagus cancer (C15), gastric cancer (C16), intestinal cancer (C18‐C20), liver cancer (C22), pancreatic cancer (C25), and lung cancer (C34), which were coded using the 10th revision of the *International Classification of Diseases* (ICD‐10).

### 2.4 Ascertainment of covariates

A smoker was defined as having smoked more than 100 cigarettes in his or her lifetime.^16^ Alcohol consumption was defined as at least one alcoholic drink per month. Physical activity was measured as the total metabolic equivalent per day for all activities. Diabetes duration was calculated by subtracting the data of first diagnosis of diabetes from the date of enrolment at baseline assessment. Education, income, medication history (antidiabetic medication, insulin, and lipid‐lowering medicine), and history of physician‐diagnosed CHD and stroke were assessed at the time of interview. Height and body weight were measured for individuals while ensuring that heavy clothes and shoes were removed before the measurements. Body mass index (BMI) was calculated by dividing weight in kilograms by the square of height in meters.

### 2.5 Statistical analysis

Baseline characteristics were described by means (SD) for continuous variables and proportions for categorical variables (*n*, %). The distribution of GGT values was right skewed, therefore a natural‐log transformed GGT was applied. GGT values were categorized into five groups using the 20th (reference), 40th, 60th, and 80th percentiles as cut‐points; the cutoff points corresponded to 17, 23, 31, and 48 U/L. Person‐time was calculated for each individual from the date of recruitment to the date of death or the date of last follow‐up (31 December 2020).

Continuous variables were compared between groups with independent *t* tests and categorical variables were compared with chi‐square tests. Cox regression models were used to analyze the associations of serum GGT concentrations with all‐cause and cause‐specific mortality. Hazard ratios (HRs) and 95% confidence intervals (CIs) were calculated according to quintiles of log‐transformed GGT concentrations. Model 1 was adjusted for age at blood draw (continuous) and sex. Model 2 was further adjusted for education, income, smoking status, alcohol consumption, BMI (continuous), physical activity (continuous), and lipid‐lowering medication use. All‐cause and CVD mortality were additionally adjusted for self‐reported stroke and CHD at baseline. Model 3 was further adjusted for diabetes duration (continuous), antidiabetic medication use and insulin use.

To assess linearity in the associations of serum GGT concentration with the risk of all‐cause and specific‐cause mortality in the fully adjusted model, we performed restricted cubic spline analysis with four knots and calculated the likelihood ratio test by comparing the model with only the linear term of these markers to the model with both the linear and cubic spline terms. The top 0.1% of GGT concentrations (*n* = 20) was truncated to avoid the effect of outliers on the curve. *p* for nonlinearity <.05 was considered nonlinearity, and *p* for linearity <.05 was considered linearity.

We also conducted stratified analysis according to age (<70, ≥70 years), sex (female, male), BMI (<25, ≥25 kg/m^2^), smoking status (never, ever), median diabetes duration (<5, ≥5 years), and dyslipidemia (no, yes). Dyslipidemia was defined by lipid indicators (total cholesterol ≥6.22 mmol/L or triglycerides ≥2.26 mmol/L or high‐density lipoprotein <1.04 mmol/L or low‐density lipoprotein ≥4.14 mmol/L) or hospital diagnosis. To investigate potential effect modification by these stratification variables, we used a likelihood ratio test comparing the models with and without interaction terms between GGT concentrations and each of the stratification variables. Considering that weight change is a typical symptom in T2DM progression, we further analyze the associations of serum GGT concentrations with all‐cause and cause‐specific mortality among nonobese (BMI <30 kg/m^2^) T2DM patients.

Sensitivity analysis was performed by excluding participants in the first 2 years of follow‐up (*n* = 556), participants who died due to accident (ICD‐10: V00‐Y98) (*n* = 153), or participants who used lipid‐lowering medicine (*n* = 2081). Additionally, considering the disruption caused by the COVID‐19 pandemic, we changed the date of the last follow‐up to 31 December 2019 (before the start of the COVID‐19 pandemic) for sensitivity analysis.

All analysis were conducted in R 4.0.5 (The R foundation for Statistical Computing, Vienna, Austria). All statistical tests were two sided, and *p* < .05 was considered statistically significant.

## 3 RESULTS

The median follow‐up period was 7.04 years (interquartile range: 6.98–7.08 years). Among 19 961 participants, 2728 deaths occurred, including 902 (33.09%) deaths due to CVD and 754 (27.58%) deaths due to cancer. The distribution of GGT is shown in Figure S1. The mean (SD) value was 40.53 (61.01) U/L for GGT and 3.40 (0.67) for log‐transformed GGT. Table 1 summarizes the main characteristics of the T2DM patients according to quintile of serum GGT concentration. At baseline, the mean (SD) age was 62.9 (9.9) years. Participants with higher GGT concentrations had a higher BMI, lower levels of physical activity, and a shorter diabetes duration. They also tended to be younger and more likely to be current smokers or drinkers.

**TABLE 1:** Baseline characteristics of the participants according to quintile of log‐transformed GGT concentration^a^.

Characteristics	Total	Quintile of log‐transformed GGT					
		Q1(≤2.83)	Q2(2.83–3.14)	Q3(3.14–3.43)	Q4(3.43–3.87)	Q5(>3.87)	*p* value
*N* (%)	19 961	4059 (20.3)	4262 (21.4)	3786 (19.0)	3877 (19.4)	3977 (19.9)	
Follow‐up time (SD), years	6.6 (1.3)	6.7 (1.2)	6.7 (1.2)	6.6 (1.3)	6.6 (1.3)	6.5 (1.4)	<.0001
Age at blood draw (SD), years	62.9 (9.9)	63.0 (10.2)	63.1 (9.6)	63.3 (9.7)	62.7 (9.6)	62.2 (10.2)	<.0001
Female, *n* (%)	12 136 (60.8)	3201 (78.9)	2850 (66.9)	2266 (59.9)	2013 (51.9)	1806 (45.4)	<.0001
Education, *n* (%)^b^							<.0001
Without formal education	10 927 (54.7)	2497 (61.5)	2396 (56.2)	2079 (54.9)	1946 (50.2)	2009 (50.5)	
Primary school	3311 (16.6)	574 (14.1)	726 (17.0)	649 (17.1)	666 (17.2)	696 (17.5)	
Middle school	3803 (19.1)	653 (16.1)	756 (17.7)	722 (19.1)	820 (21.2)	852 (21.4)	
High school and above	1851 (9.3)	322 (7.9)	375 (8.8)	330 (8.7)	421 (10.9)	403 (10.1)	
Annual household income, *n* (%)^b^, yuan							<.0001
<10 000	3102 (15.5)	678 (16.7)	675 (15.8)	601 (15.9)	571 (14.7)	577 (14.5)	
10 000–30 000	5208 (26.1)	1037 (25.5)	1098 (25.8)	1030 (27.2)	1049 (27.1)	994 (25.0)	
40 000–100 000	8901 (44.6)	1831 (45.1)	1902 (44.6)	1673 (44.2)	1729 (44.6)	1766 (44.4)	
>100 000	2641 (13.2)	497 (12.2)	560 (13.1)	458 (12.1)	505 (13.0)	621 (15.6)	
Smoking status, *n* (%)^b^							<.0001
Never	14 312 (71.7)	3356 (82.7)	3234 (75.9)	2692 (71.1)	2557 (66.0)	2473 (62.2)	
Previous	1131 (5.7)	142 (3.5)	231 (5.4)	227 (6.0)	244 (6.3)	287 (7.2)	
Current	4374 (21.9)	534 (13.2)	764 (17.9)	841 (22.2)	1050 (27.1)	1185 (29.8)	
Alcohol consumption, *n* (%)^b^							<.0001
Never	15 544 (77.9)	3644 (89.8)	3591 (84.3)	2983 (78.8)	2797 (72.1)	2529 (63.6)	
Previous	979 (4.9)	134 (3.3)	192 (4.5)	222 (5.9)	223 (5.8)	208 (5.2)	
Current	3378 (16.9)	260 (6.4)	471 (11.1)	568 (15.0)	845 (21.8)	1234 (31.0)	
Total physical activity (SD), MET hours per day	11.7 (15.1)	12.3 (15.5)	11.8 (14.2)	11.6 (15.4)	11.5 (14.8)	11.4 (15.5)	.04
BMI (SD), kg/m^2^	25.3 (3.5)	24.1 (3.3)	24.8 (3.4)	25.5 (3.4)	26.0 (3.4)	26.3 (3.5)	<.0001
Diabetes duration (SD), years	6.1 (5.7)	6.7 (6.0)	6.4 (5.8)	6.2 (5.9)	5.9 (5.4)	5.3 (5.1)	<.0001
Antidiabetic medication use (%)	13 835 (69.3)	2685 (66.1)	2888 (67.8)	2664 (70.4)	2778 (71.7)	2820(70.9)	<.0001
Insulin use (%)	3010 (15.1)	712 (17.5)	725 (17.0)	563 (14.9)	497 (12.8)	513 (12.9)	<.0001
Lipid‐lowering medication use (%)	2081 (10.4)	317 (7.8)	408 (9.6)	457 (12.1)	440 (11.3)	459 (11.5)	<.0001
Dyslipidemia (%)	9676 (48.5)	1282 (31.6)	1807 (42.4)	1915 (50.6)	2265 (58.4)	2407 (60.5)	<.0001
CHD (%)	1628 (8.2)	278 (6.8)	339 (8.0)	375 (9.9)	316 (8.2)	320 (8.0)	<.0001
Stroke (%)	1963 (9.8)	345 (8.5)	410 (9.6)	441 (11.6)	401 (10.3)	366 (9.2)	.001

Abbreviations: BMI, body mass index; CHD, coronary heart disease; GGT, gamma‐glutamyl transferase; MET, metabolic equivalent.

^a^ Mean (SD) values and percentages are reported for continuous and categorical variables, respectively.

^b^ For some variables, the totals did not sum to 100% due to small proportions of participants choosing “prefer not to answer.”

### 3.1 All‐cause mortality.

**FIGURE 1:**
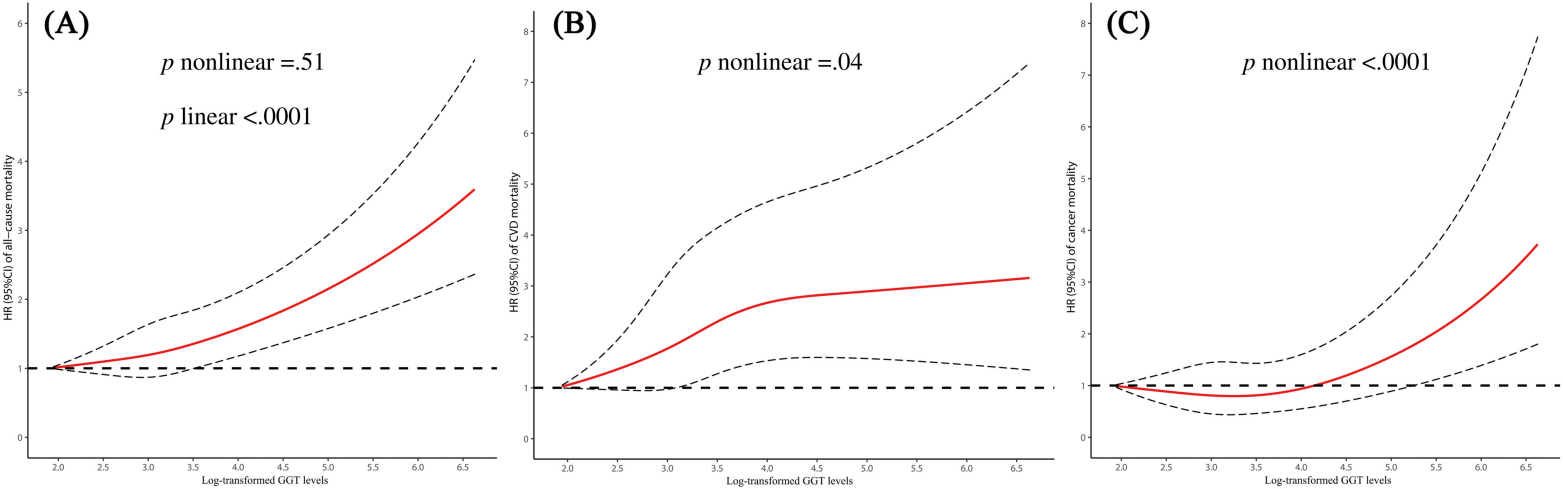
The dose–response relationship between log‐transformed GGT (A–C) concentrations and all‐cause and specific‐cause mortality according to restricted cubic spline regression analysis. The solid line represents estimates of hazard ratios; dashed lines represent 95% confidence intervals. GGT, gamma‐glutamyl transferase.

As shown in Table 2, compared to the lowest quintile (Q1), the highest quintile (Q5) of GGT concentration was related to an increased risk of all‐cause mortality in the fully adjusted multivariable models (HR = 1.63, 95% CI: 1.44–1.84). As illustrated in Figure 1, restricted cubic spline analysis showed that the association of log‐transformed GGT with all‐cause mortality was generally linear (*p* for nonlinear = .51 and *p* for linear <.0001).

**TABLE 2:** Associations of serum GGT concentration with all‐cause and cause‐specific mortality.

Cause of death	Quintile of log‐transformed GGT concentrations, HR (95% CI)^a^				
	Q1	Q2	Q3	Q4	Q5
All‐cause deaths, *N*	474	553	515	535	651
Model 1	ref	1.10 (0.97–1.24)	1.11 (0.98–1.26)	1.20 (1.06–1.36)	1.43 (1.27–1.62)
Model 2	ref	1.12 (0.99–1.26)	1.17 (1.03–1.33)	1.30 (1.14–1.47)	1.61 (1.42–1.82)
Model 3	ref	1.12 (0.99–1.26)	1.17 (1.03–1.33)	1.32 (1.16–1.50)	1.63 (1.44–1.84)
CVD deaths, *N*	130	173	185	200	214
Model 1	ref	1.27 (1.01–1.59)	1.47 (1.17–1.85)	1.68 (1.35–2.10)	1.76 (1.41–2.20)
Model 2	ref	1.23 (0.98–1.55)	1.44 (1.15–1.81)	1.68 (1.34–2.11)	1.87 (1.49–2.35)
Model 3	ref	1.23 (0.98–1.54)	1.43 (1.14–1.80)	1.68 (1.34–2.11)	1.87 (1.49–2.35)
Cancer deaths, *N*	134	147	142	136	195
Model 1	ref	0.99 (0.78–1.25)	1.02 (0.81–1.30)	0.98 (0.77–1.25)	1.37 (1.10–1.72)
Model 2	ref	1.00 (0.79–1.26)	1.06 (0.83–1.35)	1.02 (0.80–1.31)	1.44 (1.15–1.82)
Model 3	ref	0.99 (0.79–1.26)	1.05 (0.83–1.34)	1.02 (0.79–1.30)	1.43 (1.13–1.81)

Abbreviations: CI, confidence interval; CVD, cardiovascular disease; GGT, gamma‐glutamyl transferase; HR, hazard ratio; ref, reference.

^a^ Model 1 was adjusted for age at blood draw (continuous) and sex. Model 2 was further adjusted for education level (without formal education, primary school, middle school, high school and above, unknown), income (<10 000, 10 000–30 000, 40 000–100 000, ≥100 000 yuan, unknown), smoking (never, previous, current, unknown), alcohol consumption (never, previous, current, unknown), BMI (continuous), physical activity (continuous), and lipid‐lowering medicine use (no, yes, unknown). All‐cause and CVD mortality were additionally adjusted for self‐reported stroke (yes, no, unknown) and coronary heart disease (yes, no, unknown) at baseline. Model 3 was further adjusted for diabetes duration (continuous), antidiabetic medication use (yes, no, unknown), and insulin use (yes, no, unknown).

### 3.2 CVD mortality.

Higher GGT concentrations were associated with an increased risk of CVD mortality (Model 2: HR for Q5 vs. Q1 = 1.87, 95% CI: 1.49–2.35). The association remained stable after further adjustment for diabetes duration, antidiabetic medicine, and insulin use (Table 2). For CVD‐specific mortality, higher GGT concentrations were associated with a higher risk of CHD and stroke mortality (HR for Q5 vs. Q1 = 2.09, 95% CI: 1.23–3.55 and HR for Q5 vs. Q1 = 1.91, 95% CI: 1.37–2.66, respectively) (Figure S2). The dose–response relationship between GGT concentrations and CVD mortality shown in Figure 1 is nonlinear (*p* for nonlinear = .04).

### 3.3 Cancer mortality.

We observed positive associations of GGT concentration with cancer mortality (HR for Q5 vs. Q1 = 1.43, 95% CI: 1.13–1.81) (Table 2). For cancer‐specific mortality, higher GGT concentrations were associated with increased risks of liver cancer and intestinal cancer mortality (HR for Q5 vs Q1 = 4.70, 95% CI: 2.45–9.04 and HR for Q5 vs Q1 = 2.56, 95% CI = 1.00–6.51, respectively) (Figure S2). Restricted cubic spline analysis showed a nonlinear positive relationship of log‐transformed GGT concentrations with cancer mortality (*p* for nonlinear <.0001) (Figure 1).

**FIGURE 2:**
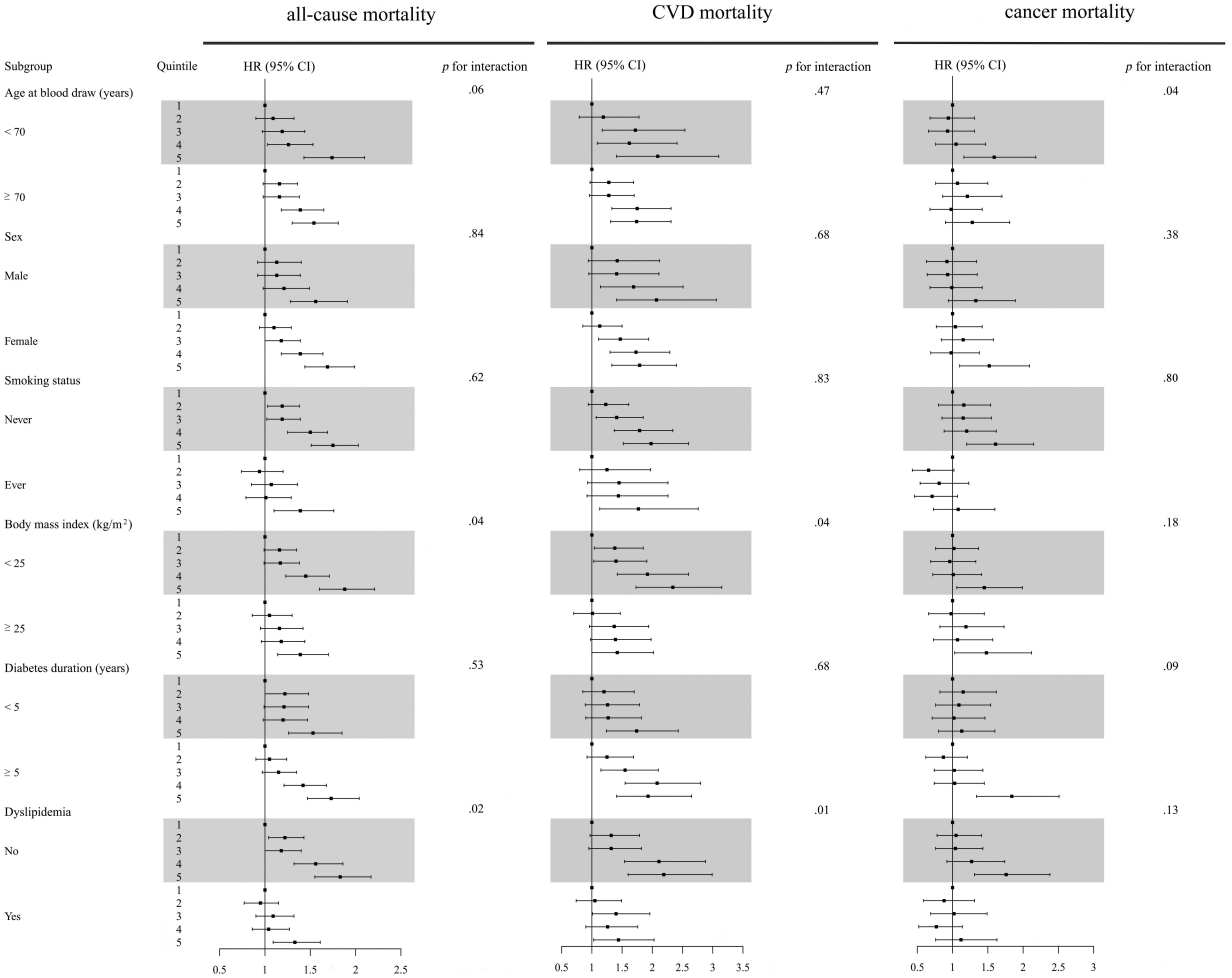
Forest plot of stratified analyses for the highest (Q5) vs the lowest quintile (Q1) of GGT concentrations and the risk of all‐cause and cause‐specific mortality. CI, confidence interval; CVD, cardiovascular disease; GGT, gamma‐glutamyl transferase; HR, hazard ratio.

Figure 2 shows the forest plot results of stratified analysis. Associations of GGT concentration with all‐cause, CVD, and cancer mortality were largely consistent across subgroups, with several exceptions. Significant interactions were observed between BMI, dyslipidemia, and GGT concentration for the risk of all‐cause and CVD mortality, with stronger positive effects for the BMI <25 kg/m^2^ group and those without dyslipidemia. (both *p* for interaction <.05). Furthermore, among nonobese T2DM patients, serum GGT concentration was also positively associated with the risk of all‐cause, CVD, and cancer mortality (Table 3). In sensitivity analysis, the aforementioned associations remained after excluding 556 participants who died within 2 years after the blood draw, 153 participants who died due to an accident, and 2081 participants who used lipid‐lowering medicine or changed the date of the last follow‐up to before the COVID‐19 pandemic. (Tables S1–S4).

**TABLE 3:** Associations of serum GGT concentration with all‐cause and cause‐specific mortality among nonobese T2DM patients.

Cause of death	Quintile of log‐transformed GGT concentrations, HR (95% CI)^a^				
	Q1	Q2	Q3	Q4	Q5
All‐cause deaths, *N*	451	521	468	487	578
Model 1	ref	1.10 (0.97–1.25)	1.11 (0.98–1.27)	1.22 (1.07–1.39)	1.45 (1.28–1.64)
Model 2	ref	1.08 (0.95–1.23)	1.09 (0.95–1.24)	1.20 (1.05–1.37)	1.45 (1.27–1.64)
Model 3	ref	1.08 (0.96–1.23)	1.09 (0.96–1.24)	1.22 (1.07–1.39)	1.47 (1.29–1.67)
CVD deaths, *N*	121	161	166	183	180
Model 1	ref	1.28 (1.01–1.63)	1.49 (1.18–1.89)	1.75 (1.39–2.21)	1.72 (1.36–2.18)
Model 2	ref	1.23 (0.97–1.56)	1.39 (1.10–1.77)	1.67 (1.32–2.11)	1.73 (1.37–2.19)
Model 3	ref	1.23 (0.97–1.55)	1.39 (1.10–1.76)	1.67 (1.32–2.11)	1.73 (1.37–2.19)
Cancer deaths, *N*	129	139	130	120	169
Model 1	ref	0.98 (0.77–1.25)	1.02 (0.80–1.30)	0.95 (0.74–1.23)	1.34 (1.06–1.69)
Model 2	ref	0.98 (0.77–1.24)	1.02 (0.80–1.30)	0.95 (0.74–1.23)	1.35 (1.06–1.71)
Model 3	ref	0.97 (0.76–1.24)	1.01 (0.79–1.29)	0.95 (0.73–1.22)	1.33 (1.05–1.68)

Abbreviations: CI, confidence interval; CVD, cardiovascular disease; GGT, gamma‐glutamyl transferase; HR, hazard ratio; ref, reference.

^a^ Model 1 was adjusted for age at blood draw(continuous) and sex. Model 2 was further adjusted for education level (without formal education, primary school, middle school, high school and above, unknown), income (<10 000, 10 000–30 000, 40 000–100 000, ≥100 000 yuan, unknown), smoking (never, previous, current, unknown), alcohol consumption (never, previous, current, unknown), BMI (continuous), physical activity (continuous), and lipid‐lowering medicine use (no, yes, unknown). All‐cause and CVD mortality were additionally adjusted for self‐reported stroke (yes, no, unknown) and coronary heart disease (yes, no, unknown) at baseline. Model 3 was further adjusted for diabetes duration (continuous), antidiabetic medication use (yes, no, unknown), and insulin use (yes, no, unknown).

## 4 DISCUSSION.

In this prospective cohort study conducted among community‐dwelling Chinese T2DM patients, we observed positive associations between GGT concentrations and all‐cause, CVD, and cancer mortality. Additionally, we found significant interactions of GGT concentrations with BMI and dyslipidemia for the risk of all‐cause and CVD mortality, suggesting that T2DM patients with BMI <25 kg/m^2^ or without dyslipidemia have a higher risk of mortality.

Some prospective studies have shown a positive relationship between GGT concentration and all‐cause mortality in the general population.^17^, ^18^, ^19^ In line with our findings, there was a positive association between serum GGT and all‐cause mortality in T2DM patients. In the UK Biobank study, GGT was independently associated with increased all‐cause mortality and liver‐related outcomes.^19^ However, some of these studies were conducted only in men,^6^, ^20^ and a population‐based cohort study in Germany reported no association between serum GGT concentration and mortality in women using any applied model.^21^ In stratified analysis, we found stronger effects in the participants with BMI <25 kg/m^2^. This may be related to the obesity paradox reported in T2DM, with lower BMI associated with a more severe disease phenotype.^22^ T2DM patients with lower BMI may be more sensitive to visceral fat accumulation, have a greater genetic predisposition to insulin resistance, or have early islet failure.^23^ Severe T2DM will lead to a reduction in fat and muscle mass and, therefore, be more reflected in BMI.^24^ This may help to explain why some studies found a link between a low BMI and a higher mortality risk. Surprisingly, we also observed that the effect between higher GGT concentrations and increased mortality was stronger in participants without dyslipidemia. Similar results were reported in a Taiwanese prospective study in which dyslipidemia was associated with a reduced risk of hepatocellular carcinoma mortality.^25^ However, dyslipidemia changes over time, and the effect of lipid‐lowering medicine on liver function needs more investigation to elucidate the underlying mechanisms.

With respect to CVD mortality, our findings suggest a positive association of GGT concentration with CVD mortality, which is consistent with some prior observational studies and meta‐analysis.^26^, ^27^ In the Framingham Heart Study, associations of GGT concentration with CVD outcome and metabolic syndrome were still robust after adjustment for traditional cardiac risk factors and C‐reactive protein.^4^ Among T2DM patients, a randomized controlled trial with 9757 participants showed that GGT >70 U/L was associated with higher death risks due to CVD.^28^ In contrast, no association has been found between higher GGT concentration with CVD mortality in the third US National Health and Nutrition Examination Survey or a Korean occupational cohort with a 7‐year follow‐up study.^5^, ^29^ An observational cohort study of 1952 T2DM patients also failed to show a relationship between GGT concentration and CVD mortality after adjusting HbA1c, metabolic syndrome, Charlson Comorbidity Index score, insulin therapy, and metformin dose.^30^ Overall, associations between GGT concentration and CVD mortality in T2DM patients remain uncertain. Studies have shown that GGT plays an important role in glutathione extracellular catabolism, and serum‐like GGT has been found in atheromatous plaques.^31^ It has been postulated that a potential mechanism linking GGT concentration and CVD risk is that GGT mediates redox reactions in the atheromatous plaque environment and may influence further development and changes in atherosclerotic plaques.^31^

Regarding cancer mortality, prior studies have suggested that higher GGT concentrations predict cancer mortality in a healthy population.^6^, ^18^, ^29^ Similar findings were also observed in the T2DM population.^28^, ^30^ In a prospective study of 79 279 Austrian men followed up for 19 years, the authors found that GGT concentrations were consistently associated with malignancies of digestive organs, the respiratory system, and urinary organs.^32^ Furthermore, a case‐cohort study based on Taiwanese men indicated that GGT concentration was significantly associated with hepatocellular carcinoma mortality.^20^ In our analysis, GGT concentrations were associated with liver cancer and intestinal cancer. The function of GGT plays a role in regulating oxidative stress and the balance of cellular proliferation and apoptosis.^33^ GGT concentrations are significantly elevated in malignant or premalignant lesions and have been considered a factor that confers survival and growth for rapidly dividing tumor cells.^34^ In stratified analysis, we found stronger effects in participants aged <70 years. One possible explanation is that the ability to maintain homeostasis and the liver's ability to clear xenobiotics progressively are diminished in elderly individuals.^35^ Because GGT is a marker for oxidative stress, the oxidative damage hypothesis is one of the most prominent theories for aging. Almost all organisms manifest physiological functional loss as they age.^35^

Recent studies have shown that GGT is also an important factor affecting the gut microbial structure and gut microbiota diversity is closely related to our health.^36^ The disturbances in gut microbiota are involved in the progression of T2DM, obesity, and cirrhosis.^37^, ^38^, ^39^ Results of a study conducted in patients with metabolic syndrome showed the structural and functional alterations in the gut microbiome of male patients with elevated GGT, characterized by reduced gut microbiota diversity, increased inflammation‐related “harmful bacteria” and reduced anti‐inflammatory and antiobesity‐related “beneficial bacteria”.^36^ GGT, as a proven biomarker of oxidative stress, may mediate the chronic inflammatory process in metabolic syndrome. In addition, GGT is also a direct marker that may reflect the extent of hepatic fat deposition, which may be a major cause of obesity.^40^

Currently, two potential biological explanations for the association between GGT and mortality are more frequently suggested. One is that GGT directly contributes to the increased risk of mortality, which may be due to the fact that GGT is a marker of oxidative stress, which plays an important role in CVD and cancer progression.^11^, ^41^ Another is that the positive association may be mediated by NAFLD, which causes a high prevalence of liver damage in T2DM population. Studies have demonstrated a strong association of obesity and increased hepatic lipid with insulin resistance and cardiometabolic risk.^42^, ^43^ This is an essential issue in the field; however, consensus about the mechanisms involved has not yet been achieved.

Our findings suggested that GGT may be a powerful indicator of T2DM prognosis. Given the role of GGT in multiple disease mechanisms, we hypothesize GGT could be a simple, safe, and noninvasive diagnostic tool that could be used as a complement to traditional diagnostic methods. Our study provided additional evidence for the prediction of GGT on T2DM prognosis, which may provide a new direction for clinically targeted therapy. However, it may be premature to apply GGT in clinical practice.

A major strength of the study is that research population comprised T2DM patients in the Chinese community‐dwelling population, who differ from patients in a hospital (possible Berkson's bias), and they were thus well represented. In addition, serum GGT detection was completed in the same laboratory with unified methods and instruments, effective controls of measurement bias. However, several limitations need to be considered. First, although patients with known hepatic disease were excluded, the prevalence of NAFLD was unknown due to a lack of ultrasound liver examination, which did not allow us to evaluate the role of NAFLD in the associations. Second, there may be regression dilution bias using the baseline GGT concentration, so the one‐time collection of baseline information may not enable us to assess changes throughout the whole follow‐up period. Third, the present study lacked data on the time of smoking initiation and how much was smoked as well as changes in income levels during COVID‐19, which may have an effect on mortality. Finally, the mortality surveillance system may underreport and misclassify, though underreporting surveys are performed every year for the database, with automatic evaluation of misclassifications. Misclassification and underreporting were <5%.

## 5 CONCLUSION

In conclusion, our findings suggest that, in the Chinese T2DM population, elevated serum GGT concentration is associated with higher mortality for all‐cause, CVD, and cancer. Future studies should focus on revealing the role of obesity, NAFLD, and lipids in this association.

## AUTHOR CONTRIBUTIONS

Conception and design of the study: Ming Wu, Jian Su, Haoyu Guan, Jinyi Zhou, Chong Shen. Collection of data: Hao Yu, Yu Qin, Zheng Zhu, Jie Yang, Enchun Pan, Yan Lu, Jinyi Zhou. Data analysis, interpretation of results, and writing of the manuscript: Haoyu Guan, Ke Liu, Xikang Fan, Jian Su. All the authors revised the manuscript critically for its intellectual content and approved the final version.

## FUNDING INFORMATION

This work was supported by grants from Jiangsu Province Leading Talents and Innovation Team Program (No. K201105); Jiangsu Provincial Fifth “333 Project” (BRA2020090); Jiangsu Provincial Health Commission 2020 Medical Research Project Approval (M2020085); the Suzhou Program of Science & Technology Development (SS202010).

## CONFLICT OF INTEREST STATEMENT

The authors declare that they have no conflicts of interest.

## ETHICS STATEMENT

Ethics approval for this study was granted by the Ethics Board of Jiangsu Provincial Center for Disease Control and Prevention (No. 2013026). All participants provided written informed consent, and the methods were carried out in accordance with relevant guidelines and regulations.

## Supporting information


**Data S1.** Supplementary File
