# Supplementary material for: Association of gamma‐glutamyl transferase concentrations with all‐cause and cause‐specific mortality in Chinese adults with type 2 diabetes
Source: J Diabetes. 2023 May 9;15(8):674–84. doi: 10.1111/1753-0407.13399 (PMC10415869; doi:10.1111/1753-0407.13399)
Supplement: Supplementary file 1 — Data S1. Supplementary File [file JDB-15-674-s001.docx]

| Supplementary Table 1. Sensitivity analyses for excluding deaths within the first 2 years of follow-up (n = 556) for the associations between serum GGT concentrations and risk of all-cause and cause-specific mortality | | | | | |
| --- | --- | --- | --- | --- | --- |
| Cause of Death | Quintile of log-transformed GGT concentrations, HR(95%CI)a | | | | |
|  | Q1 | Q2 | Q3 | Q4 | Q5 |
| All-cause deaths, N | 375 | 445 | 515 | 535 | 651 |
| Model 1 | ref | 1.12 (0.98-1.29) | 1.11 (0.98-1.26) | 1.20 (1.06-1.36) | 1.43 (1.27-1.62) |
| Model 2 | ref | 1.14 (0.99-1.31) | 1.17 (1.03-1.33) | 1.30 (1.14-1.47) | 1.61 (1.42-1.82) |
| Model 3 | ref | 1.14 (0.99-1.31) | 1.17 (1.03-1.33) | 1.32 (1.16-1.50) | 1.63 (1.44-1.84) |
| CVD deaths, N | 99 | 144 | 185 | 200 | 214 |
| Model 1 | ref | 1.39 (1.08-1.80) | 1.47 (1.17-1.85) | 1.68 (1.35-2.10) | 1.76 (1.41-2.20) |
| Model 2 | ref | 1.35 (1.04-1.74) | 1.44 (1.15-1.81) | 1.68 (1.34-2.11) | 1.87 (1.49-2.35) |
| Model 3 | ref | 1.35 (1.04-1.74) | 1.43 (1.14-1.80) | 1.68 (1.34-2.11) | 1.87 (1.49-2.35) |
| Cancer deaths, N | 100 | 113 | 142 | 136 | 195 |
| Model 1 | ref | 1.03 (0.78-1.34) | 1.02 (0.81-1.30) | 0.98 (0.77-1.25) | 1.37 (1.10-1.72) |
| Model 2 | ref | 1.03 (0.78-1.34) | 1.06 (0.83-1.35) | 1.02 (0.80-1.31) | 1.44 (1.15-1.82) |
| Model 3 | ref | 1.02 (0.78-1.34) | 1.05 (0.83-1.34) | 1.02 (0.79-1.30) | 1.43 (1.13-1.81) |
| Abbreviations: GGT: gamma-glutamyl transferase; HR, hazard ratio; CI, confidence interval; CVD, cardiovascular disease; ref, reference.  aModel 1 was adjusted for age at blood draw(continuous) and sex. Model 2 was further adjusted for education level (without formal education, primary school, middle school, high school and above, unknown), income (<10000, 10000-30000, 40000-100000, ≥100,000 yuan, unknown), smoking (never, previous, current, unknown), alcohol consumption (never, previous, current, unknown), BMI (continuous), physical activity (continuous), and lipid-lowering medicine use (no, yes, unknown). All-cause and CVD mortality were additionally adjusted for self-reported stroke (yes, no, unknown) and CHD (yes, no, unknown) at baseline. Model 3 was further adjusted for diabetes duration (continuous), antidiabetic medication use (yes, no, unknown), and insulin use (yes, no, unknown). | | | | | |

| Supplementary Table 2. Sensitivity analyses for excluding accidental deaths (n = 153) for the associations between serum GGT concentrations and risk of all-cause and cause-specific mortality | | | | | |
| --- | --- | --- | --- | --- | --- |
| Cause of Death | Quintile of log-transformed GGT concentrations, HR(95%CI)a | | | | |
|  | Q1 | Q2 | Q3 | Q4 | Q5 |
| All-cause deaths, N | 447 | 511 | 495 | 508 | 614 |
| Model 1 | ref | 1.08 (0.95-1.23) | 1.13 (0.99-1.29) | 1.21 (1.06-1.38) | 1.45 (1.28-1.64) |
| Model 2 | ref | 1.10 (0.97-1.25) | 1.19 (1.04-1.35) | 1.30 (1.14-1.48) | 1.62 (1.42-1.84) |
| Model 3 | ref | 1.10 (0.97-1.25) | 1.19 (1.04-1.35) | 1.33 (1.16-1.51) | 1.64 (1.44-1.87) |
| CVD deaths, N | 130 | 173 | 185 | 200 | 214 |
| Model 1 | ref | 1.27 (1.01-1.60) | 1.47 (1.17-1.84) | 1.68 (1.34-2.10) | 1.77 (1.42-2.21) |
| Model 2 | ref | 1.24 (0.99-1.56) | 1.44 (1.14-1.80) | 1.68 (1.34-2.11) | 1.88 (1.50-2.36) |
| Model 3 | ref | 1.23 (0.98-1.55) | 1.43 (1.14-1.79) | 1.68 (1.34-2.11) | 1.88 (1.50-2.36) |
| Cancer deaths, N | 134 | 147 | 142 | 136 | 195 |
| Model 1 | ref | 0.99 (0.78-1.25) | 1.02 (0.80-1.29) | 0.98 (0.77-1.25) | 1.38 (1.10-1.73) |
| Model 2 | ref | 1.00 (0.79-1.27) | 1.06 (0.83-1.34) | 1.02 (0.80-1.31) | 1.45 (1.15-1.83) |
| Model 3 | ref | 1.00 (0.79-1.26) | 1.05 (0.83-1.34) | 1.02 (0.79-1.30) | 1.44 (1.14-1.82) |
| Abbreviations: GGT: gamma-glutamyl transferase; HR, hazard ratio; CI, confidence interval; CVD, cardiovascular disease; ref, reference.  aModel 1 was adjusted for age at blood draw(continuous) and sex. Model 2 was further adjusted for education level (without formal education, primary school, middle school, high school and above, unknown), income (<10000, 10000-30000, 40000-100000, ≥100,000 yuan, unknown), smoking (never, previous, current, unknown), alcohol consumption (never, previous, current, unknown), BMI (continuous), physical activity (continuous), and lipid-lowering medicine use (no, yes, unknown). All-cause and CVD mortality were additionally adjusted for self-reported stroke (yes, no, unknown) and CHD (yes, no, unknown) at baseline. Model 3 was further adjusted for diabetes duration (continuous), antidiabetic medication use (yes, no, unknown), and insulin use (yes, no, unknown). | | | | | |

| Supplementary Table 3. Sensitivity analyses for excluding lipid-lowering medicine use (n = 2081) for the associations between serum GGT concentrations and risk of all-cause and cause-specific mortality | | | | | |
| --- | --- | --- | --- | --- | --- |
| Cause of Death | Quintile of log-transformed GGT concentrations, HR(95%CI)a | | | | |
|  | Q1 | Q2 | Q3 | Q4 | Q5 |
| All-cause deaths, N | 427 | 486 | 443 | 477 | 573 |
| Model 1 | ref | 1.09 (0.96-1.24) | 1.13 (0.99-1.29) | 1.22 (1.07-1.39) | 1.44 (1.26-1.63) |
| Model 2 | ref | 1.10 (0.97-1.26) | 1.18 (1.03-1.35) | 1.33 (1.16-1.52) | 1.61 (1.41-1.83) |
| Model 3 | ref | 1.10 (0.97-1.26) | 1.19 (1.04-1.36) | 1.35 (1.18-1.55) | 1.63 (1.42-1.85) |
| CVD deaths, N | 109 | 149 | 150 | 169 | 180 |
| Model 1 | ref | 1.32 (1.03-1.70) | 1.51 (1.18-1.94) | 1.74 (1.36-2.22) | 1.80 (1.41-2.29) |
| Model 2 | ref | 1.27 (0.99-1.63) | 1.47 (1.14-1.89) | 1.75 (1.37-2.24) | 1.89 (1.47-2.42) |
| Model 3 | ref | 1.27 (0.99-1.62) | 1.47 (1.14-1.88) | 1.75 (1.37-2.24) | 1.88 (1.47-2.42) |
| Cancer deaths, N | 126 | 129 | 124 | 126 | 179 |
| Model 1 | ref | 0.94 (0.73-1.20) | 1.00 (0.78-1.29) | 1.00 (0.78-1.28) | 1.38 (1.09-1.74) |
| Model 2 | ref | 0.95 (0.74-1.21) | 1.04 (0.81-1.33) | 1.05 (0.81-1.35) | 1.47 (1.16-1.87) |
| Model 3 | ref | 0.94 (0.74-1.21) | 1.03 (0.80-1.33) | 1.04 (0.81-1.34) | 1.45 (1.14-1.85) |
| Abbreviations: GGT: gamma-glutamyl transferase; HR, hazard ratio; CI, confidence interval; CVD, cardiovascular disease; ref, reference.  aModel 1 was adjusted for age at blood draw(continuous) and sex. Model 2 was further adjusted for education level (without formal education, primary school, middle school, high school and above, unknown), income (<10000, 10000-30000, 40000-100000, ≥100,000 yuan, unknown), smoking (never, previous, current, unknown), alcohol consumption (never, previous, current, unknown), BMI (continuous), physical activity (continuous), and lipid-lowering medicine use (no, yes, unknown). All-cause and CVD mortality were additionally adjusted for self-reported stroke (yes, no, unknown) and CHD (yes, no, unknown) at baseline. Model 3 was further adjusted for diabetes duration (continuous), antidiabetic medication use (yes, no, unknown), and insulin use (yes, no, unknown). | | | | | |

| Supplementary Table 4. Sensitivity analyses for the follow-up until December 31, 2019 for the associations between serum GGT concentrations and risk of all-cause and cause-specific mortality | | | | | |
| --- | --- | --- | --- | --- | --- |
| Cause of Death | Quintile of log-transformed GGT concentrations, HR(95%CI)a | | | | |
|  | Q1 | Q2 | Q3 | Q4 | Q5 |
| All-cause deaths, N | 392 | 444 | 426 | 447 | 543 |
| Model 1 | ref | 1.06 (0.92-1.21) | 1.10 (0.96-1.26) | 1.19 (1.04-1.37) | 1.42 (1.24-1.62) |
| Model 2 | ref | 1.09 (0.95-1.24) | 1.18 (1.03-1.36) | 1.32 (1.15-1.52) | 1.63 (1.42-1.87) |
| Model 3 | ref | 1.09 (0.95-1.25) | 1.18 (1.03-1.36) | 1.35 (1.17-1.55) | 1.65 (1.44-1.89) |
| CVD deaths, N | 105 | 135 | 143 | 162 | 178 |
| Model 1 | ref | 1.21 (0.93-1.56) | 1.39 (1.08-1.79) | 1.65 (1.29-2.11) | 1.76 (1.38-2.25) |
| Model 2 | ref | 1.19 (0.92-1.54) | 1.40 (1.08-1.80) | 1.70 (1.33-2.19) | 1.94 (1.51-2.49) |
| Model 3 | ref | 1.18 (0.91-1.53) | 1.38 (1.07-1.79) | 1.70 (1.32-2.18) | 1.92 (1.49-2.47) |
| Cancer deaths, N | 118 | 115 | 121 | 117 | 163 |
| Model 1 | ref | 0.87 (0.67-1.13) | 0.98 (0.76-1.27) | 0.95 (0.74-1.24) | 1.29 (1.01-1.64) |
| Model 2 | ref | 0.89 (0.69-1.15) | 1.04 (0.8-1.34) | 1.01 (0.78-1.32) | 1.39 (1.08-1.79) |
| Model 3 | ref | 0.89 (0.69-1.15) | 1.03 (0.8-1.34) | 1.01 (0.78-1.32) | 1.38 (1.08-1.78) |
| Abbreviations: GGT: gamma-glutamyl transferase; HR, hazard ratio; CI, confidence interval; CVD, cardiovascular disease; ref, reference.  aModel 1 was adjusted for age at blood draw(continuous) and sex. Model 2 was further adjusted for education level (without formal education, primary school, middle school, high school and above, unknown), income (<10000, 10000-30000, 40000-100000, ≥100,000 yuan, unknown), smoking (never, previous, current, unknown), alcohol consumption (never, previous, current, unknown), BMI (continuous), physical activity (continuous), and lipid-lowering medicine use (no, yes, unknown). All-cause and CVD mortality were additionally adjusted for self-reported stroke (yes, no, unknown) and CHD (yes, no, unknown) at baseline. Model 3 was further adjusted for diabetes duration (continuous), antidiabetic medication use (yes, no, unknown), and insulin use (yes, no, unknown). | | | | | |

**
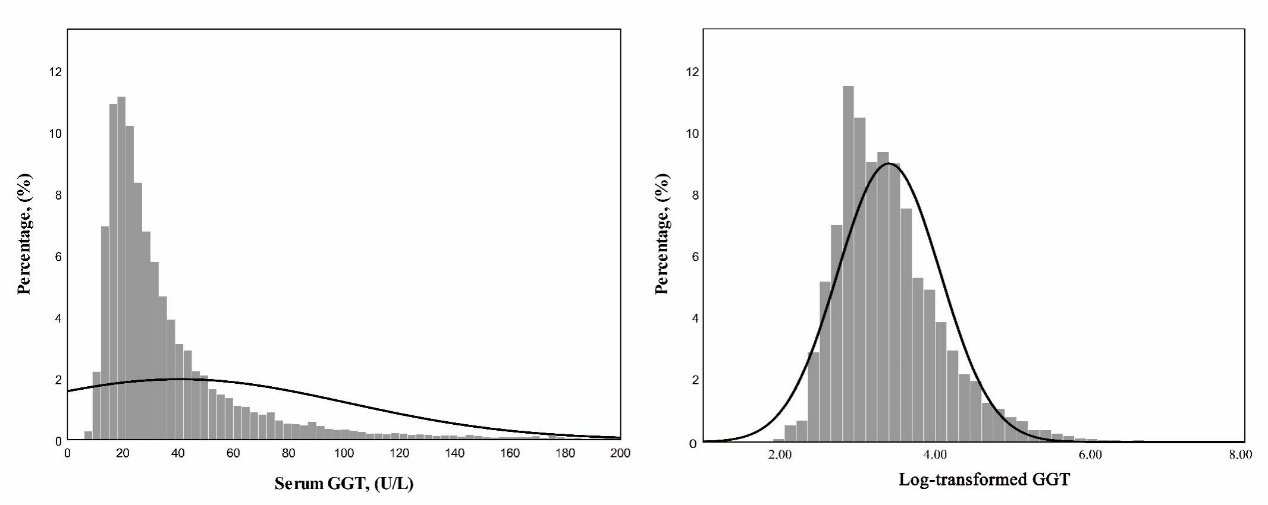
**Supplementary Figure 1. The distributions of original and Log-transformed GGT concentrations


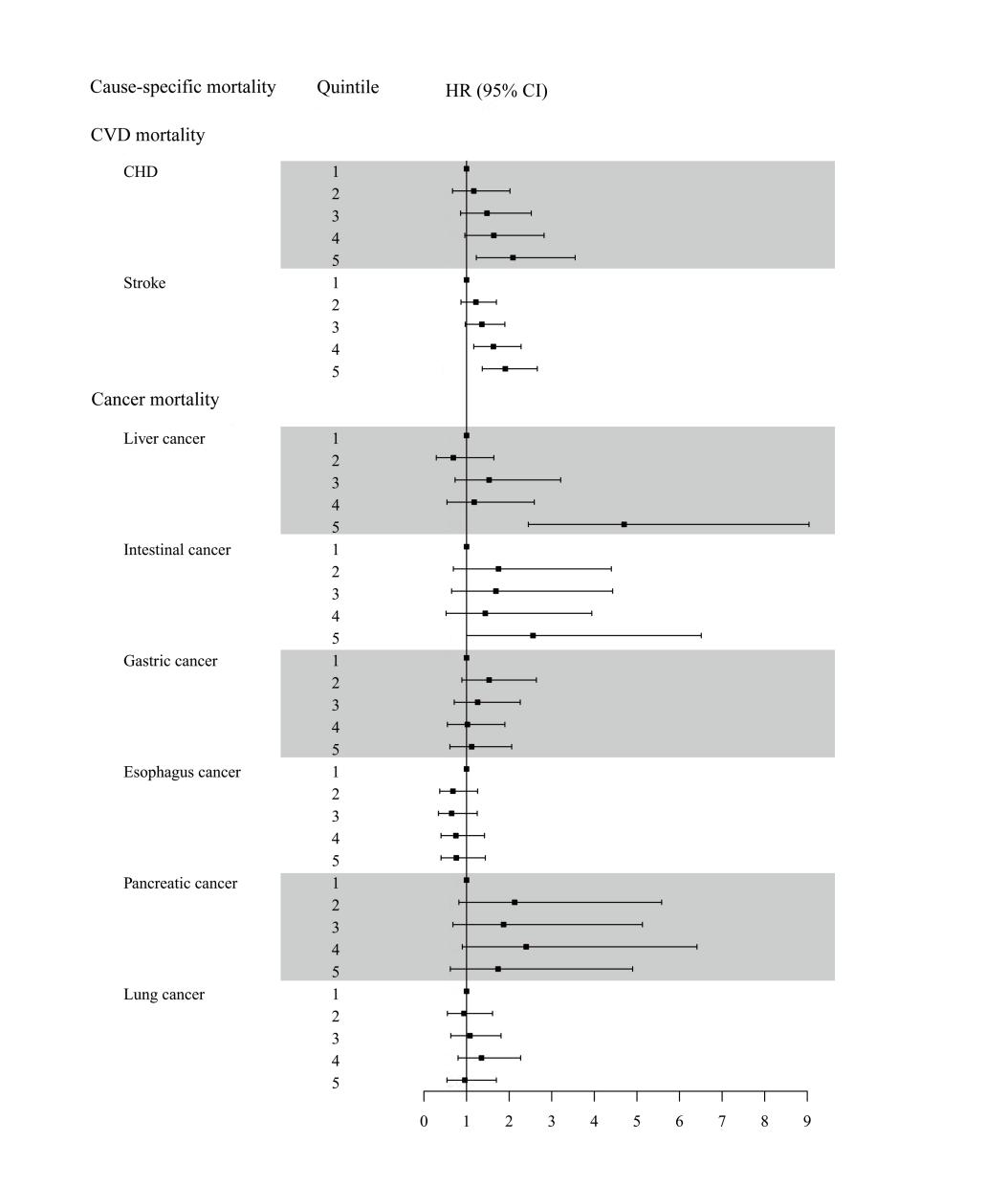
Supplementary Figure 2. Forest plots of the associations between serum GGT concentrations and the risk of cause-specific mortality based on the fully adjusted cox regression models.
